# Virus-specific CD8+ T cells infiltrate melanoma lesions and retain function despite high PD-1 expression

**DOI:** 10.1186/2051-1426-3-S2-O6

**Published:** 2015-11-04

**Authors:** Dan A Erkes, Toktam Mohgbeli, Christopher M Snyder

**Affiliations:** 1 Thomas Jefferson University, Philadelphia, PA, USA

## 

It is well known that CD8+ T cell infiltration of tumors is correlated with positive patient prognosis. Because of this, many viral vaccine vectors have been created to induce expansion and infiltration of tumor-specific CD8+ T cells. Cytomegalovirus (CMV) is a herpes virus that induces a persistent/latent infection for the life of the host. It is able to drive large populations of viral-specific T cells that are fully functional and infiltrate most tissues. We have established that acute infection with a recombinant murine CMV virus expressing an altered gp100 antigen (MCMV-gp100) caused expansion and tumor infiltration of gp100-specific CD8+ T cells, leading to a therapeutic delay in the growth of B16 melanomas in mice. Coincidentally, we observed that at least 20% of the CD8+ tumor infiltrating lymphocytes (TIL) were MCMV-specific. These virus-specific T cells infiltrated tumors independently of viral replication. Unexpectedly, T cell infiltration of tumors occurred even in latently infected animals, long after viral replication had been controlled. Together, these data suggest that virus-specific CD8+ T cells can become TIL, even long after the primary infection. We were curious about differences between tumor and virus-specific CD8+ TIL. As expected, after acute MCMV-gp100 infection, gp100-specific CD8+ TIL were PD-1^hi^ and markedly dysfunctional. Virus-specific CD8+ T cells were also PD-1^hi^ in the tumor after acute MCMV-gp100 infection, but these cells remained fully functional. PD-1 is an activation marker expressed on recently stimulated CD8+ T cells and both gp100-specific and virus-specific T cells expressed PD-1 after acute MCMV-gp100 infection. In the circulation of tumor-bearing mice or in the absence of a tumor, both gp100-specific and virus-specific CD8+ T cells lost PD-1 expression over time. However, PD-1^hi^ cells that migrated into tumors retained expression of PD-1 regardless of specificity. Strikingly, introducing B16 tumors into MCMV-gp100 latently infected mice after PD-1 expression was lost, resulted in PD-1 up-regulation on and subsequent dysfunction of gp100-specific T cells that entered the tumor, but not virus-specific T cells found in the same tumors. Together, these data suggest that the tumor environment sustains PD-1 expression on recently activated CD8+ TIL independently of antigen in the tumor, but that the presence of antigen in the tumor drives subsequent CD8+ TIL dysfunction.

